# Assessment of childhood short stature: a GP guide

**DOI:** 10.3399/bjgp23X732525

**Published:** 2023-03-31

**Authors:** Helen L Storr, Joseph Freer, Jenny Child, Justin H Davies

**Affiliations:** Centre for Endocrinology, William Harvey Research Institute, Barts and the London School of Medicine, Queen Mary University, London; chair, British Society of Paediatric Endocrinology Growth Disorders Special Interest group (BSPED GD-SIG).; Wolfson Institute of Population Health, Queen Mary University of London, London.; BSPED GD-SIG and Global Registry for Novel Therapies in Rare Bone & Endocrine Conditions (GloBE-REG); patient advisor, Genomics England.; Regional Centre for Paediatric Endocrinology, Southampton Children’s Hospital, Faculty of Medicine, University of Southampton, Southampton; co-chair, BSPED GD-SIG.

## INTRODUCTION

Childhood short stature may be an early indicator of chronic illness, psychosocial deprivation, or may be a normal variant.^1^^,^^2^ In England, 2% of 4–5-year-olds are short for their age and sex (height below 2nd centile). Prevalence is strongly linked to poverty; in England, the most deprived areas have twice the prevalence of short stature compared with the least deprived.^3^ It is critical that primary care providers are able to accurately assess a child’s growth when they encounter short children or when assessing chronic illness. It is helpful to recognise that currently girls and ethnic minority groups with short stature are less likely to be referred, assessed, and treated despite their greater need.^4^^,^^5^ It is crucial that clinicians recognise their implicit biases and understand the social disparities in the assessment of childhood short stature to improve timely diagnosis, referral, and early access to treatment.^6^ This article outlines how to undertake a targeted assessment in a child presenting with short stature.

## WHAT ARE THE MAIN INFLUENCES ON HEIGHT DURING CHILDHOOD?

**Figure 1.**
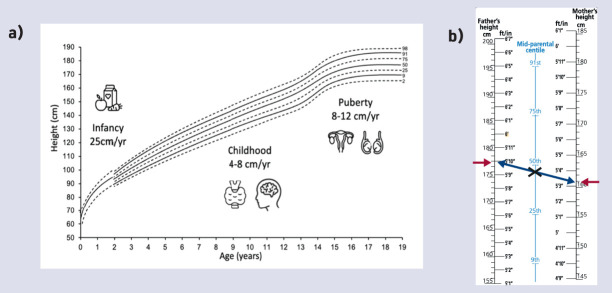
*Main determinant of height during phases of childhood growth. a) Human linear growth can be divided into three phases: infancy, childhood, and pubertal growth. Infancy growth is dependent on nutrition and the growth rate (height velocity (HV) in cm/year) is rapid (up to 25 cm/year) but declines as childhood growth supervenes. Childhood growth is predominant from about 3 years of age with a steady HV between 4‒8 cm/yr. This phase is more dependent on hormones (growth hormone and thyroid hormone). At puberty, there is a growth spurt with a dramatic increase in height velocity dependent on growth hormone and sex hormones. This is approximately 2 years earlier in girls (mean age 12 years) and coincides with early breast development. In boys, puberty is well established at the start of the growth spurt (peak HV mean age 14 years). b) Boys’ UK growth chart 2–18 years parent height comparator. Mother’s and father’s heights are plotted on their respective scales and the two points are joined with a line. The mid-parental centile is where the line crosses the centile line in the middle and can be compared with the child’s current height centile. Ninety per cent of children’s height centiles are within ± 2 centile spaces of the mid-parental height/centile and 99% of children are within 3 centile spaces of their mid-parental height/centile. Reproduced from  the Royal College of Paediatrics and Child Health (https://www.rcpch.ac.uk/sites/default/files/Boys_2-18_years_growth_chart.pdf).*

Appreciation of the main influences on height at different ages is helpful to direct enquiry to the cause of short stature. In the first 2–3 years, nutrition is the predominant influence on length/height. Although adequate nutrition is essential for growth until adult height, growth hormone and thyroid hormone are the main determinants from around 3 years to puberty. However, during puberty, sex hormones (oestrogen and testosterone) and growth hormone supervene (Figure 1a).^7^

## WHEN SHOULD A CHILD PRESENTING WITH CONCERNS ABOUT HEIGHT BE EVALUATED?

Four scenarios merit consideration: 1) a child presenting with symptoms of chronic illness; 2) a child presenting with a height less than the 2nd centile; 3) a child with a height more than 3 centile spaces below that expected for their parents’ heights; and 4) a child with growth failure (poor growth rate/height velocity), that is, drop in height of more than 1 centile on the growth chart. Importantly, the child’s height may not be below the 2nd centile in the latter two scenarios.

## EVALUATION OF CHILDHOOD SHORT STATURE

The commonest causes are familial short stature and constitutional delay of growth and puberty (CDGP) (Box 1).^1^^,^^8^ Awareness of these two distinct non-pathological growth patterns is important to avoid unnecessary anxiety and investigation. Other causes can be broadly grouped as primary or secondary causes (Box 1).^1^

**Box 1.** Causes of childhood short stature

	***Normal variant short stature***	
Familial short stature	Within 3 centile spaces of their mid-parental height (MPH)^a^ No red flags	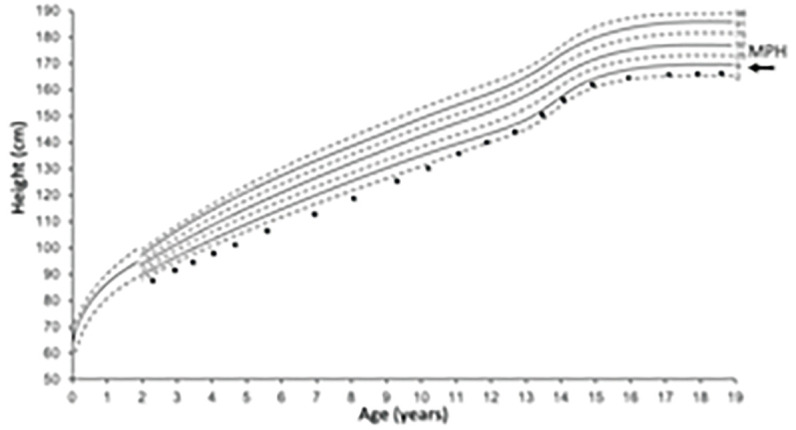
Constitutional delay of growth and puberty	Short stature for age and sex Normal growth rate Delayed puberty and puberty growth spurt^b^	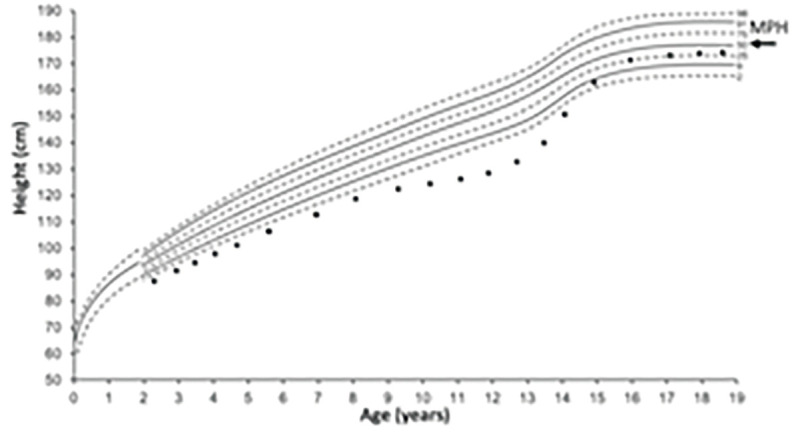
	***Pathological causes of short stature***	
**Primary**	**Secondary**	
Secondary to being born small for gestational age with no catch-up in height growth^c^	Nutritional insufficiency, for example, malabsorption, poor intake, and feeding problems	
Skeletal dysplasia (usually associated with body disproportion)	Systemic diseases/inflammation, for example, Crohn’s disease	
Chromosomal disorders, for example, Turner syndrome in girls	Psychosocial deprivation	
	Endocrine disorders, for example, hypothyroidism and growth hormone deficiency	

a *Ninety-nine per cent of children are within 3 centile spaces of their mid-parental height/centile (and 90% within ± 2 centile spaces). Note — one very short parent may indicate a genetically inherited problem.*

b *Boys present twice as often as girls and a family history of constitutional delay of growth and puberty (CDGP) is common. Delayed skeletal maturation (bone age calculated from non-dominant hand and wrist X-ray) is observed.*

c *Consider in children presenting over the age of 4 years with a height below the 2nd centile AND with a birth weight or length less than 2nd centile.*

**Box 2.** Red flags in short stature assessment

**Red flags in clinical assessment**	**Red flags in height assessment**
Weight loss, constipation, diarrhoea, vomiting, headaches, abnormal fat stores, disproportion, dysmorphic features, and anaemia	Height <0.4th centile for age and sex OR Height <2nd centile for age and sex with other red flags
Features of hypothyroidism or other chronic illness	Height centile >3 centile spaces below mid-parental height (MPH) centile
No signs of puberty in girls by age 13 and boys by age 14 years	Drop in height of >1 centile spaces

The cornerstones of assessment in primary care are a targeted history, examination, accurate growth measurement(s) (requiring appropriate, calibrated equipment), and parental height assessment. The clinician should be cognisant that girls and children from ethnic minority groups are often overlooked in the assessment of short stature.^1^^,^^8^

## ESSENTIAL POINTS IN THE HISTORY

- Ethnic background.
- Birth weight/gestation.
- Neurocognitive development and behaviour.
- Family history of short stature, delayed puberty, and consanguinity.
- Features of chronic illness, for example, weight loss, poor appetite, gastrointestinal symptoms, fatigue, and recurrent infections.
- Headaches, visual problems, nausea, and vomiting (space-occupying lesion of the brain).
- Medications, for example, steroids (oral/topical/inhaled).
- Psychosocial or safeguarding concerns.

## IMPORTANT FEATURES ON EXAMINATION

- Height and weight.
- Head circumference in infants.
- Cardiovascular examination, for example, heart murmur.
- Features of hypothyroidism.
- Features of dysmorphism or disproportion (short arms/legs).

## HOW SHOULD LENGTH/HEIGHT BE ACCURATELY ASSESSED?

Length should be measured from age 0‒2 years (using a calibrated infant measuring device) and standing height measured after 2 years (using a manual or electronic wall-mounted stadiometer).^9^ For length, the child’s head is held against the fixed board and the infant’s legs are straight. The correct position should be adopted for standing height measurements with feet flat and heels against the wall (shoes removed). The child’s head should be straight and looking straight ahead (the Frankfort horizontal plane).^9^ Measurement equipment should be regularly cleaned/maintained according to the manufacturer recommendations. Height/length should be plotted on appropriate growth charts to the nearest 0.1 cm. Gestational correction should be used for infants born <37 weeks (until 1 year for infants born 32‒36 weeks and 2 years for infants born <32 weeks).^8^

## OTHER HELPFUL ASSESSMENTS

- *Mid-parental height (MPH)* is calculated based on the parental height measurements and represents a child’s expected height as an adult. Comparing the MPH centile to the child’s current height centile provides insight into whether the child’s growth is proceeding as expected and can identify familial short stature. A child with a height more than 3 centile spaces below the MPH centile will require assessment and consideration of referral as they are more likely to have a growth disorder. Measured parent height measurements are preferred but reported estimates are acceptable. Different methods calculate MPH/MPH centile^2^ including using 1) the parent height comparator on the UK growth charts (Figure 1b) or 2) the following equation:
MPH (girl ) (cm)= Mother's height (cm)+Father'sheight(cm) -13cm/2 
MPH (boy) (cm) = Mother's height (cm)+Father'sheight(cm)-13cm/2
- *Height velocity (growth rate)*: if there are concerns, height should be tracked over time (6‒12 months apart). A drop in more than 1 height centile may indicate a growth disorder.

## WHAT BASIC INVESTIGATIONS CAN BE DONE IN PRIMARY CARE?

Basic testing to rule out common primary or secondary growth disorders includes:^1^^,^^8^

- FBC and ESR (inflammation and anaemia);
- renal profile;
- liver function;
- calcium, phosphate, and alkaline phosphatase (renal and calcium disorders);
- coeliac screen; and
- thyroid function (TSH and FT4).

## TAKE-HOME MESSAGES

- Accurate evaluation of height is critical in the assessment of child health.
- Disparities in the assessment, referral, investigation, and treatment of short children disadvantages girls and ethnic minority groups, and primary care teams have a vital role in reducing these inequalities.
- Poor childhood height growth may reflect psychosocial problems, social inequalities, or can be an indicator of underlying disease (often preceding other symptoms and signs).
- Familial short stature is common and benign, and can be distinguished from pathological short stature by accurate child and parental height assessment, and by demonstrating a normal growth rate.
- Delayed diagnosis of treatable disorders presenting with early faltering height growth is common, limiting treatment options and leading to worse clinical outcomes.
- Poor measurement technique or inaccurate interpretation of height data leads to unnecessary anxiety, misdiagnosis, and wasted resources.
